# Detecting age-related changes in skeletal muscle mechanics using ultrasound shear wave elastography

**DOI:** 10.1038/s41598-023-47468-z

**Published:** 2023-11-16

**Authors:** Filiz Ateş, Justus Marquetand, Manuela Zimmer

**Affiliations:** 1 Institute of Structural Mechanics and Dynamics in Aerospace Engineering, University of Stuttgart, Stuttgart, Germany; 2 Department of Epileptology, Hertie-Institute for Clinical Brain Research, University of Tübingen, Tübingen, Germany; 3 Department of Neural Dynamics and Magnetoencephalography, Hertie-Institute for Clinical Brain Research, University of Tübingen, Tübingen, Germany; 4 MEG-Center, University of Tübingen, Tübingen, Germany

**Keywords:** Biomedical engineering, Diagnostic markers, Predictive markers, Prognostic markers, Biomaterials

## Abstract

Aging leads to a decline in muscle mass and force-generating capacity. Ultrasound shear wave elastography (SWE) is a non-invasive method to capture age-related muscular adaptation. This study assessed biceps brachii muscle (BB) mechanics, hypothesizing that shear elastic modulus reflects (i) passive muscle force increase imposed by length change, (ii) activation-dependent mechanical changes, and (iii) differences between older and younger individuals. Fourteen healthy volunteers aged 60–80 participated. Shear elastic modulus, surface electromyography, and elbow torque were measured at five elbow positions in passive and active states. Data collected from young adults aged 20–40 were compared. The BB passive shear elastic modulus increased from flexion to extension, with the older group exhibiting up to 52.58% higher values. Maximum elbow flexion torque decreased in extended positions, with the older group 23.67% weaker. Significant effects of elbow angle, activity level, and age on total and active shear elastic modulus were found during submaximal contractions. The older group had 20.25% lower active shear elastic modulus at 25% maximum voluntary contraction. SWE effectively quantified passive and activation-dependent BB mechanics, detecting age-related alterations at rest and during low-level activities. These findings suggest shear elastic modulus as a promising biomarker for identifying altered muscle mechanics in aging.

## Introduction

With advancing age, skeletal muscles undergo changes that detriment their quality due to a decline in muscle mass and force-generating capacity^1^. The condition is defined as sarcopenia which is associated with inactivity, altered neural control of muscles, hormonal and metabolic changes, inflammatory mediators, and inadequate protein intake^2,3^. The loss of muscle strength linked to a decreased number of motoneurons and fibers, reduced fiber size, and alterations of muscle architecture leads to a decrease in mobility and an increased risk of falls^4^. Therefore, in vivo monitoring of changes in muscle properties with aging and early detection of sarcopenia symptoms in community settings is critical for eliminating some of the risks and premature decline in our ever-aging society. However, available evaluation tools such as body composition measurements do not allow us to assess the mechanics of individual muscles. For only a limited number of muscles, mechanical properties have been collected either using direct invasive force measurements^5–7^ or e.g. minimally-invasive intramuscular pressure evaluations^8–10^. Consequently, there is a need for an in vivo and non-invasive system that could specify the mechanical characteristics of individual muscles.

Ultrasound shear wave elastography (SWE) quantifies local mechanical properties by measuring the propagation velocity of the shear waves traveling within the tissue. The shear wave velocity measured from skeletal muscles was shown to be directly associated with stiffness^11^ and the tensile loads applied on muscle tissue^12^. SWE has demonstrated the capability to represent both passive^13,14^ and active muscle mechanics^14–16^, making it potential tool for monitoring and early detection of muscular alterations in aging individuals. While earlier animal studies have suggested that aging muscles exhibit increased stiffness primarily due to e.g., collagen accumulation^17^ and alterations in the extracellular matrix^18^, recent investigations using ultrasound elastography have produced a range of findings. Some studies have reported lower stiffness for BB^19^, rectus femoris^20,21^, gastrocnemii^20^, and hamstring^19^ muscles of older adults at rest. In contrast, other studies have found no significant differences in the shear modulus of gastrocnemii^22,23^, vastus intermedius^24^, and soleus^20^, and increased values for BB^25^ particularly at the most extended elbow position, vastus lateralis, and rectus femoris^26^ at extended knee positions in older individuals. Only a few studies have investigated the active muscle properties and reported decreased stiffness for rectus femoris and vastus intermedius in older groups^24^. Given the diversity and, at times, contrasting results, there is a critical need for a comprehensive assessment of the potential contribution and limitations of the SWE approach in monitoring age-related changes in muscle passive and active characteristics.

The objective of this study was to investigate the use of SWE in older individuals comprehensively for a better understanding of age-related changes in skeletal muscle. For this purpose, we assessed the mechanics of the biceps brachii (BB) muscle of older individuals in passive and active states with the hypotheses that shear elastic modulus reflects (i) the increase in passive muscle forces imposed by muscle length change, (ii) the activation-dependent mechanical changes in BB, and (iii) the differences between the mechanics of muscles of older and young groups.

## Results

### Anthropometrics

Participants’ average upper arm length and circumference were 31.43 ± 2.58 cm and 30.00 ± 2.87 cm, respectively. The elbow angle had a significant effect on the BB length (*p* < 0.001) but not on CSA (*p* = 0.963) (Table 1). Post-hoc tests located the differences at the muscle lengths between 60° and 120°, 150°, 180° (*p* < 0.001 for all), 90° and 150° (*p* = 0.003), 180° (*p* < 0.001) elbow angles. For all participants, the BB length at 60° was the shortest and the average length ratio (normalized to the BB length at 60°) was 1.21, 1.39, 1.48, and 1.59 for 90°, 120°, 150°, and 180° elbow angles respectively.

**Table 1:** Biceps brachii muscle anthropometrics measured using B-mode ultrasound.

Elbow angle	60°	90°	120°	150°	180°
CSA (cm^2^)	9.59 ± 3.15	9.72 ± 2.88	9.81 ± 2.80	9.89 ± 2.98	9.08 ± 3.65
BB perimeter (cm)	13.51 ± 1.87	13.40 ± 1.74	13.09 ± 1.51	13.07 ± 1.52	12.88 ± 2.06
BB length (cm)	9. 87 ± 1.90	11.74 ± 1.75	13.51 ± 1.82*	14.36 ± 1.68 *^/^**	15.39 ± 1.91 *^/^**

Values are given as mean ± standard deviation. *BB* Biceps brachii muscle, *CSA* cross-sectional area. Marked values with * and ** indicate significant differences from the length measured at 60° and 90° elbow angles, respectively.

### Characteristics of biceps brachii muscle in passive state

The normalized sEMG amplitude of BB during rest was above 1%, 3% and 5% for 56 (80%), 28 (40.0%) and 17 (24.3%) of the 70 trials, respectively.

**Figure 1:**
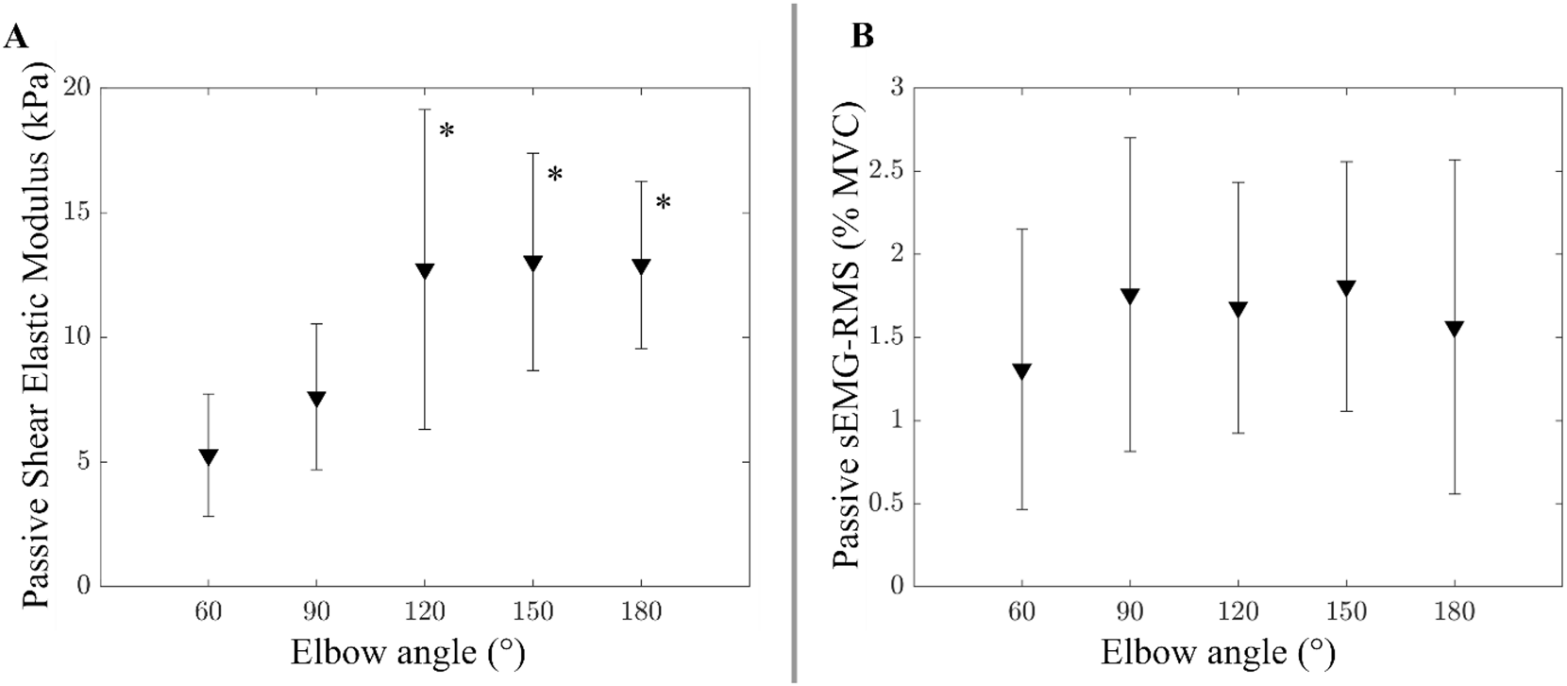
(**A**) Shear elastic modulus and (**B**) Surface electromyography amplitude of the biceps brachii muscle at passive state for different elbow angles from flexion to extension. Error bars visualize the standard deviation. * indicates that it is significantly different from the values measured at 60° (*p* ≤ 0.01).

Elbow angle had a significant effect on the passive shear elastic modulus of BB (*p* < 0.001) (Fig. 1A). Post-hoc tests located the differences between 60° and 120° (*p* = 0.005), 150° (*p* = 0.002), and 180° (*p* = 0.001) angles. No significant effects of elbow angle on passive sEMG (*p* = 0.759 for BB, *p* = 0.999 for TB) were found (Fig. 1B).

### Characteristics of biceps brachii muscle in active state

**Figure 2:**
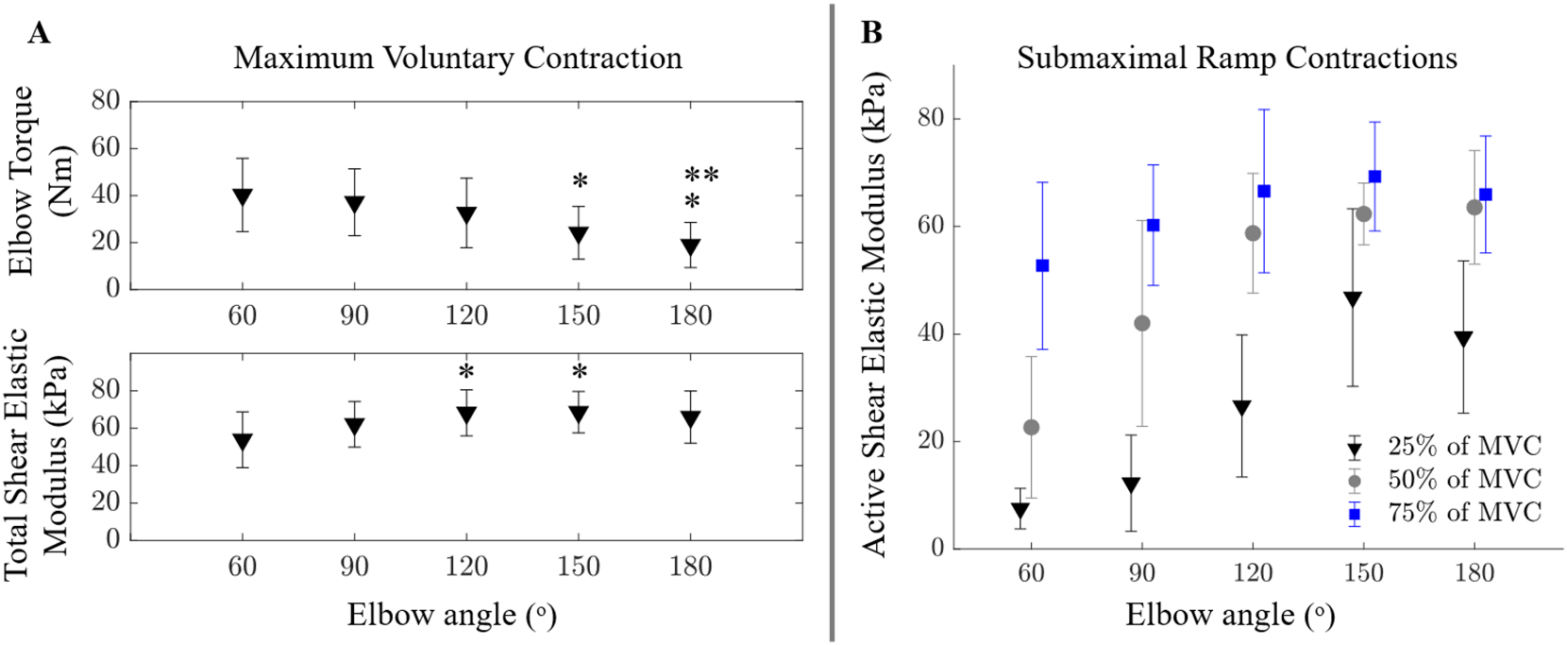
(**A**) Elbow flexion torque (top) and shear elastic modulus (bottom) during maximum voluntary contraction (MVC). * and ** indicate significant differences between them and the values measured at 60° and 90° elbow angles, respectively. (**B**) Active shear elastic modulus with respect to elbow angles tested during isometric ramp contractions at 25%, 50%, and 75% MVC. Both elbow angle (*p* < 0.001) and contraction intensity (*p* < 0.001) had significant effects on active shear elastic modulus (two-way ANOVA). Post-hoc tests located the differences between the values measured (i) at 60° and 90° (*p* = 0.006), 120°, 150°, 180° (*p* < 0.001 for all), at 90° and 120°, 150°, 180° (*p* < 0.001 for all) as well as at 120° and 150° (*p* = 0.038) elbow angles, and (ii) between all contraction intensity levels (*p* < 0.001). Error bars show the standard deviation.

*MVC:* Maximum elbow extension torque at 120° was 21.52 ± 6.37 Nm. Elbow flexion torque at MVC decreased (from 40.25 ± 15.61 N at 60° to 18.95 ± 9.60 N at 180°) with increasing elbow angle (*p* < 0.001) (Fig. 2A. Upper Panel). Post-hoc tests located the differences between 60° and 150° (*p* = 0.030), 180° (*p* = 0.001), and between 90° and 180° (*p* = 0.009) elbow angles (Fig. 2A). A significant effect of elbow angle was observed for the total shear elastic modulus (*p* = 0.021) with differences between 60° and 120° (*p* = 0.046) and 150° (*p* = 0.037) angles (Fig. 2A. Lower Panel). No significant effects were found for the active shear elastic modulus (*p* = 0.577) and sEMG amplitude of the BB (*p* = 0.398) and TB (*p* = 0.875) measured. During elbow extension contraction, the BB sEMG amplitude was 17.83% ± 9.81% MVC and during elbow flexion contractions, the average TB sEMG amplitude over the elbow angles tested was 97.67% ± 69.17% MVC.

*Submaximal ramp contractions:* Only ten out of 420 trials were excluded due to the mean absolute percentage deviation (MAPD) > 10% and the included trials matched the given torque level with no apparent deviation (Table 2). The average MAPD (over elbow angles) was 2.48%, 3.41%, and 4.49% for 25%, 50%, and 75% MVC, respectively. Two-way ANOVA showed significant effects of elbow angle (*p* < 0.001) and contraction intensity (*p* < 0.001) for both total and active shear elastic modulus (Fig. 2B). Post-hoc tests located the differences between the values measured (i) at 60° and 90° (for total *p* < 0.001; for active *p* = 0.006), 120°, 150°, 180° (*p* < 0.001 for all), at 90° and 120°, 150°, 180° (*p* < 0.001 for all) as well as at 120° and 150° (for total *p* = 0.028; for active *p* = 0.038) elbow angles, and (ii) between all contraction intensity levels (*p* < 0.001).

**Table 2:** Normalized elbow moment (%) for each contraction intensity and elbow angle as group average and standard deviation.

Elbow angle	60°	90°	120°	150°	180°
25% MVC	25.88 ± 1.94	26.08 ± 1.27	26.38 ± 1.73	27.09 ± 1.57	27.61 ± 1.22
50% MVC	49.47 ± 1.55	49.64 ± 1.82	50.03 ± 1.55	50.28 ± 1.76	52.02 ± 2.44
75% MVC	72.08 ± 2.49	74.19 ± 2.02	73.98 ± 1.73	72.01 ± 2.66	75.07 ± 3.03

The contraction intensity significantly affected the sEMG amplitude of BB and TB (*p* < 0.001). Differences were significant between all contraction intensities (post-hoc tests, *p* < 0.001 for both muscles). The elbow angle significantly affected the sEMG amplitude of BB (*p* = 0.011) but not TB (*p* = 0.173). Differences in BB sEMG were significant between 60° and 150° (post-hoc tests, *p* = 0.041).

### Differences between young and older individuals

*Anthropometrics:* The body height (*p* = 0.098) and weight (*p* = 946) of the older group were not significantly different compared to the young group^27^ whereas the average length (by 10.48% ± 1.95%, *p* < 0.001) of BB muscle over the elbow angles tested were smaller in the older group. BB CSA (*p* = 0.775) and BB length ratio (BB length normalized to 60° elbow angle, *p* = 0.087) did not show any significant differences between groups.

**Figure 3:**
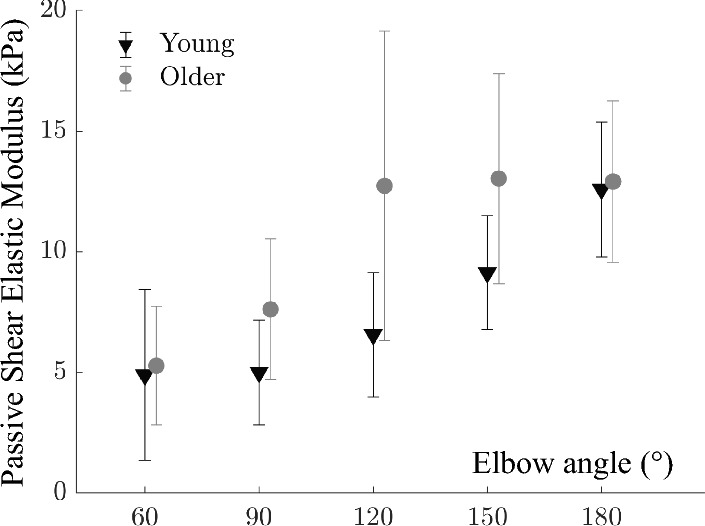
Shear elastic modulus of the biceps brachii muscle collected from young and older group at passive state for different elbow angles from flexion to extension. Grey circles and black triangles show older and young groups, respectively. The shear elastic modulus was significantly higher for the older group (*p* < 0.001). Error bars visualize the standard deviation.

*Passive state:* Two-way ANOVA (factors age and elbow angle) showed that BB passive shear elastic modulus changes both with elbow angle (*p* < 0.001) and age (*p* < 0.001) (Fig. 3). Significant interactions (*p* = 0.005) were located only at 120°. Compared to the young group, the passive shear elastic modulus was 7.92%, 52.58%, 42.78%, and 2.62% higher for older individuals at 60°, 90°, 150°, and 180° elbow angles, respectively.

*MVC:* Older group generated 25.06% less torque during maximum elbow extension at 120° (*p* = 0.034). Maximum elbow flexion torque was on average 23.67% less for older individuals (*p* < 0.001) and it decreased with increasing elbow angle (*p* < 0.001 for both age and elbow angle, no significant interactions; *p* > 0.05).

Co-contractions of BB during elbow extension MVC and co-contraction of TB during elbow flexion MVC obtained from older individuals were not significantly different compared to the young group (*p* = 0.301 and *p* = 0.478, respectively).

Two-way ANOVA (factors age and elbow angle) showed that BB total (*p* = 0.171) and active (*p* = 0.728) shear elastic modulus during MVC does not change with the elbow angle however, the factor age had a significant effect on total (*p* = 0.006) but not on active (*p* = 0.084) shear elastic modulus. No significant interactions (*p* > 0.05) were found. Total shear elastic modulus during MVC was on average 11.21% higher for the older group.

*Submaximal ramp contractions:* The performance of older individuals in keeping the torque at the desired level was inferior compared to the young group: The MAPD was on average 53.43%, 44.65%, and 28.07% higher for the older group at 25%, 50%, and 75% MVC, respectively (*p* < 0.001 for all).

**Figure 4:**
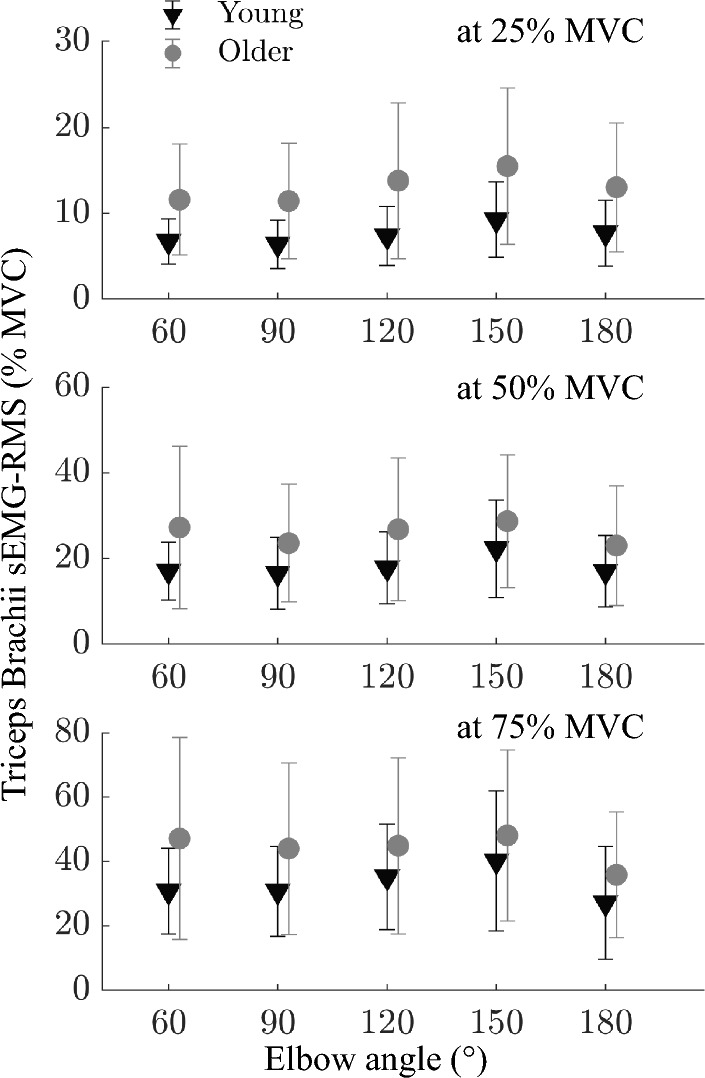
Triceps Brachii muscle surface EMG-RMS values (normalized to their maximum values measured at elbow extension MVC at 120° elbow angle) showing co-contraction during 25%, 50%, and 75% of MVC elbow flexion. Grey circles and black triangles show older and young groups, respectively. The co-contraction was significantly higher for the older group (*p* < 0.001). Error bars visualize the standard deviation.

Unlike MVCs, sEMG amplitude was significantly different between the young and older groups for TB for all intensities (*p* < 0.001, *p* = 0.001, *p* = 0.006 for 25%, 50%, 75% MVC respectively, Fig. 4) with no significant effects of elbow angle (*p* > 0.05 for all intensities).

**Figure 5:**
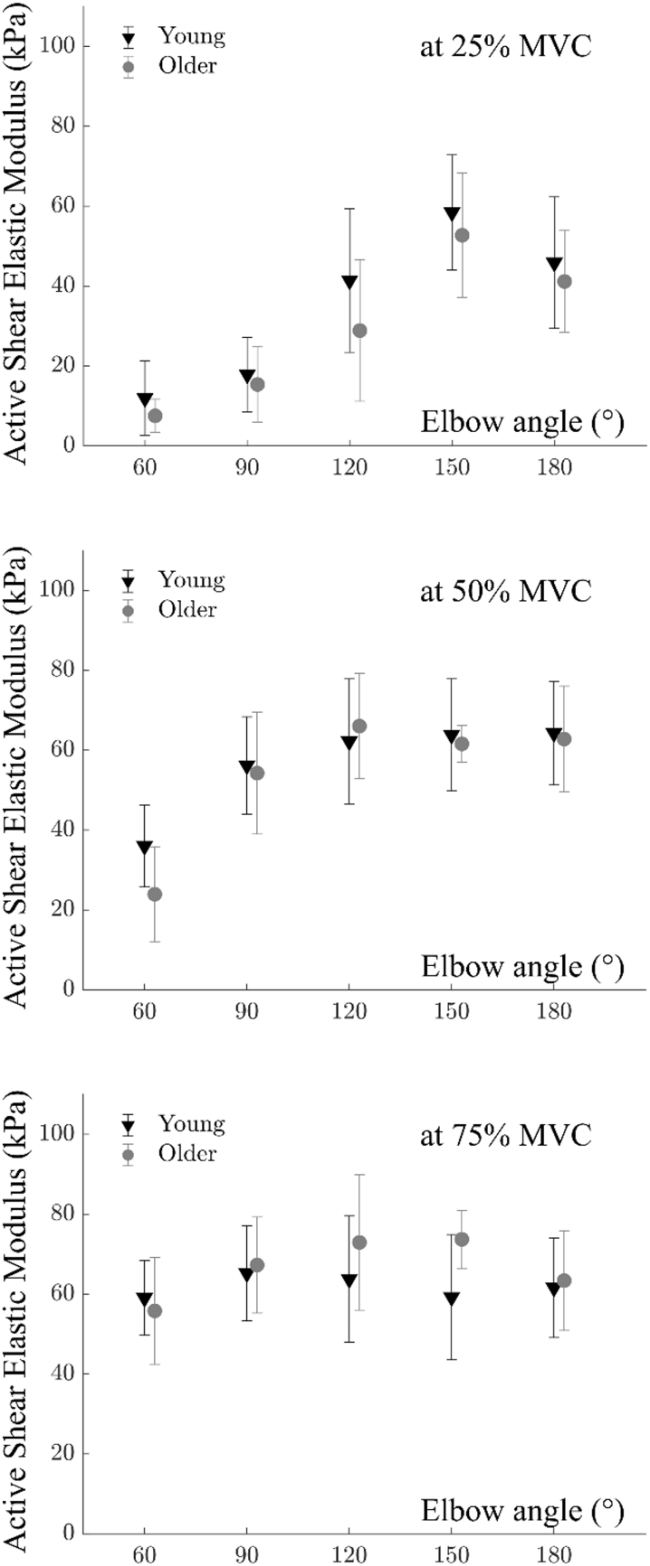
Active shear elastic modulus of the biceps brachii muscle of the older group (grey circles) at 25%, 50%, and 75% of maximum voluntary contraction (MVC) torque for the elbow angles studied (60°, 90°, 120°, 150°, and 180°) compared to the young group (black triangles) reported previously^27^. The elbow angle significantly affected active shear elastic modulus for 25% and 50% MVC (*p* < 0.001 for both, *p* = 0.101 for 75% MVC). Age had a significant effect on active shear elastic modulus for 25% MVC (*p* = 0.037) with no significant interactions (*p* > 0.05). Error bars visualize the standard deviation.

Two-way ANOVA (factors elbow angle and age for each contraction intensity) showed significant effects of elbow angle for total shear elastic modulus at all intensities (*p* < 0.001 for all) whereas, for active shear elastic modulus, angle effect was significant only for 25% (*p* < 0.001) and 50% MVC (*p* < 0.001). Age had significant effects only at 25% MVC for total (*p* = 0.006) and active (*p* = 0.037) shear elastic modulus with no significant interactions (*p* > 0.05) (Fig. 5). The total and active shear elastic modulus of BB measured from the older group was 16.18% and 20.25% lower at 25% MVC.

## Discussion

The present findings support the hypotheses that SWE can follow the alterations in the BB muscle’s passive and active properties led by changes in muscle length and elbow joint strength for older individuals. We also found that in vivo mechanics of muscles of older people and age-related adaptation can be monitored using SWE. Furthermore, the impact of increasing muscle length imposed by joint position at rest on shear elastic modulus of BB for older individuals is consistent with the literature^14,28–30^ at different populations. However, the effects of aging in passive and active conditions necessitate further detailed elaborations.

The anthropometric measures of the older population fall within the normal, non-obese range^31–34^ and the BB muscle strength evaluated by the MVC elbow torque was comparable to other studies with older participants^35–38^. Even though the height and BMI of the young and older groups were not different, the BB muscle of the older group had shorter lengths. Sarcopenia, the age-associated loss of strength, is thought to be a combination of disuse atrophy and changes in muscle architecture^4^. Earlier studies on the architecture of aging skeletal muscle reported a decrease in fascicle length and pennation angle. Since BB is not a pennate but a fusiform muscle, we expect a change only in fascicle length which directly refers to the muscle length. Fascicle lengths of the gastrocnemius^39^ and vastus lateralis^40^ were reported to drop by 10% and 14% with age, respectively. Similar to the literature, presently found BB muscle length difference between the young and older group (10.3%) might be a symptom of mild sarcopenia that led to shorter muscle lengths. There is a lack of studies conducted on aging-related alterations in muscles crossing the elbow joint. However, it can be reasonably inferred that sarcopenia impacts the BB muscle and other elbow flexors and extensors. The present finding of 20–25% lower torque-generating capacity for elbow flexion and extension for older individuals supports this anticipation. Unlike our observation, a recent study found a drop in BB CSA due to sarcopenia but it was significant only for women^41^. Reduction in muscle CSA is usually associated with a decrease in the size of type II (fast-twitch) fiber since type I (slow-twitch) fiber sizes were reported to remain unchanged with aging^42,43^. Remarkably, most of the earlier studies were performed on leg muscles (e.g.^42,44^). Supporting our finding on CSA, in contrast to vastus lateralis, BB muscle was reported to preserve its type II fibers in the older population^45^. Nevertheless, it appears that muscle size adaptation may demonstrate variant strategies between muscles, prediction of reduction in muscle mass due to aging should not be generalized, and more studies are needed for different muscles and muscle compartments for better understanding.

Accompanying muscle shortening, we found up to 53% higher passive shear elastic modulus for the older group. An increase in passive shear elastic modulus with age was previously reported for BB^25,46^ and gastrocnemius muscles^47^ and it indicates stiffening of muscle with age. This aligns with previous studies reporting enhanced extracellular matrix^18,48^ and connective tissues^17,43^, and thicker but more compliant tendons in older individuals^49,50^. These factors contribute to muscles becoming more rigid over time. However, some studies have reported lower passive stiffness for the quadriceps, hamstrings, BB^19^, and tibialis anterior muscles^51^ of older individuals, which were attributed to muscle weakness. These studies measured the muscles (except the quadriceps) at their shortest lengths^19^, which might not capture the full extent of the changes since muscle fibers, as well as connective tissues, get stiffer, in particular, at longer muscle lengths where alterations in material properties might become more pronounced and discernible. In contrast, our study investigated the effects at various muscle lengths corresponding to all joint positions, providing a more comprehensive result. It is important to develop measurement protocols that allow detecting mechanical adaptation using SWE, as higher shear elastic modulus indicating enhanced muscle stiffness can limit joint range of motion, leading to increased metabolic costs and decreased mobility in older people^52,53^. Another aspect of experimental design pertains to controlling muscle inactivity during rest. There is no consensus regarding the definition of passive state in terms of EMG amplitude level, with variations from 1 to 10% of EMG-RMS at MVC. While some studies omit EMG control, others do not disclose their threshold for absence of activity (e.g.^30^). For instance, Le Sant et al.^54^ recommended a 1% threshold for passive shear elastic modulus analysis in the triceps surae muscle group in young, healthy participants during passive stretching. In our study, however, applying a 1% threshold for exclusion would result in the loss of 80% of our data, which is impractical in any research context. For the older population and our focus on the BB muscle, we propose that a 3% threshold is a feasible alternative.

Unlike the young group, more variance for passive shear elastic modulus at elbow extension and a non-significant drop at 180° elbow angles were noticeable in the older group (Fig. 1A). We observed that keeping the elbow at extended joint positions is slightly more uncomfortable compared to flexed positions, in particular, for older individuals having stiffer and shorter muscles. The variance across different elbow angles was not attributed to changes in the activation levels of BB as the EMG amplitude remained consistent regardless of the angle (Fig. 1B). It is plausible that to relax BB in elbow extension, participants might be using other synergists and antagonists which would cause variances as well as a drop in BB resistance. This increased variability may be attributed to individual differences in the aging process, such as variations in muscle composition or the extent of muscle atrophy. This observation should be tested in bigger sample sizes to reveal whether it becomes statistically meaningful. If it holds, the finding should be addressed together with aging-related problems in neuromuscular control^55^ (see also muscle quality discussion below).

Similar to the young group^27^, the maximum elbow flexion torque was produced at 60º elbow angle for the older group and decreased at extended joint positions. However, the shear elastic modulus of BB measured during MVC did not reflect the effects of muscle length or anticipated age-associated force reduction. Notably, the higher total shear elastic modulus observed in the older group was not present when the passive component was subtracted. Therefore, the latter was due to the increase in passive muscle stiffness. This suggests that using SWE recordings during MVCs is not feasible for examining the effects of muscle length or aging. This discrepancy could be attributed to a methodological limitation, where SWE may have constraints in detecting changes in rapid and short contractions, such as MVCs. Alternatively, this could be due to the fact that the resulting elbow joint torque does not necessarily represent each muscle’s individual force production. Thus, even though the maximum force production of the joint decreases with aging, the BB muscle's high force production capacity might not change correspondingly.

Significantly, our study found that measurements during submaximal contractions provided more insights into the length effects of BB muscle. Shear elastic modulus vs elbow angle characteristics obtained during 25% and 50% MVC demonstrated these effects clearly (Fig. 5). It is reasonable to assume that the maximum activity of BB muscle is reached well before the maximum elbow torque is achieved. Therefore, contractions around 50% MVC may coincide with the maximum activity of BB. This hypothesis needs to be tested with e.g. intraoperative experiments by collecting muscle force–elbow angle data (e.g.^56–58^). If confirmed, our findings suggest that active shear elastic modulus-elbow angle curves obtained during submaximal contractions might be sufficient for the mechanical characterization of the BB in active state.

More importantly, we found that, at 25% MVC, the active shear elastic modulus was lower for older individuals compared to the young group. It is well-documented that the number of motor units drops by 40–50% in the older population (e.g.^59^), and this is one of the main reasons for reduced active force production. However, for BB muscle, it was not clear in what way the fast-twitch fibers are affected. The present finding supports the expectation discussed above that the number of fast twitch fibers of BB muscle may not be decreasing at the ages included in the present study: Motor unit recruitment is known to occur based on Henneman’s size principle^60^. The activation thresholds of the slow-twitch fibers which are also small in size are smaller; they are activated at low levels of activities compared to the fast-twitch fibers. The size principle was shown to be persistent in older ages as well^61^. Since the age-associated differences became detectable at low-level contractions using SWE, they may not be attributed to the reduction in the fast-twitch fibers. Further research is needed to explore the relationship between muscle fiber types, motor unit activity with aging, and shear elastic modulus. If the lower active shear elastic modulus is indeed attributed to changes in motor unit recruitment, supported by motor unit identification data, it would significantly enhance the value of SWE measurements. Nevertheless, our findings suggest that SWE can effectively quantify the effects of aging on the active mechanics of the BB muscle during low-level elbow torque production, corresponding to low to medium levels of muscular activities.

With aging, changes in the neuromuscular system are predicted to cause motor unit numbers to decrease secondary to motor neuron loss. Presently observed (I) enhanced difficulties to keep muscles at rest e.g. (i) 40% of the resting trials having > 3% maximum sEMG amplitude, (ii) larger variance in passive shear elastic modulus at the extended elbow positions (Fig. 1A), (II) decreased steadiness during ramp contractions e.g. (i) significantly higher mean absolute percentage deviation (MAPD) and (ii) elbow angle effects on sEMG amplitude for the older group, and (III) higher co-contraction indicate some motor control issues. These could be ascribed to the decreased neural drive due to aging^55^. Motor control problems are often associated with the coactivation of antagonists which serves to maintain joint stability as a compensation for the weakness in agonistic muscles^62,63^. The reported increase in co-contraction in older individuals may also be related to alterations in the muscle firing sequence and a delayed onset of agonistic muscle activation^8^. Coactivation designates alterations in load sharing strategies between antagonistic as well as synergic muscles. Even though, we did not find such an increase in co-contraction during MVCs, our study revealed elevated co-contraction during submaximal contractions (Fig. 4). Notably, these findings align with our SWE results, suggesting that alterations in neural control may be more pronounced at lower activation levels in BB muscle. It is possible that age-related changes in electrical properties due to neuromuscular adaptation play a substantial role in shaping mechanical properties. Nevertheless, further investigations are warranted to establish better associations between neural adaptation and load-sharing with mechanical properties.

In conclusion, the present study shows that muscular changes of BB can be detected using SWE in passive state and during low-level isometric activities therefore, SWE provides a measure of muscle quality in older individuals. Ultrasound measurements are non-invasive and radiation-free hence, there is no contraindication to using SWE in a community setting. With further research, it can be improved as a biomarker for sarcopenia assessment to prevent premature decline, frailty, and fall risk in older individuals. Following muscle stiffness as a measure can be an important target for rehabilitation and physiotherapy. Moreover, the onset of many neurological, neurodegenerative, and neuromuscular diseases occurs late in life. In near future, SWE will be used in the follow-up routine for some neurological diseases (e.g.^29,64,65^). In this case, the well-documented effects of aging on muscle stiffness will serve as a control to better differentiate the aging effects from the prognosis of the diseases.

## Methods

### Participants

14 healthy volunteers aged between 60 and 80 years old (8 females, age: 68.71 ± 5.08 years, body mass index (BMI): 26.10 ± 3.52kg/m^2^, body weight: 77.64 ± 15.78 kg, body height: 171.86 ± 10.32 cm, all data given as mean ± standard deviation) gave written informed consent and participated in the present study. All volunteers reported to perform regular physical activities such as bicycling, moderate running, hiking, going for walks or attending gymnastic classes for older adults.

For age comparison, the data sets collected in identical conditions from young adults aged between 20 and 40 years old (14 healthy volunteers, 7 females; age: 28.07 ± 5.06, BMI: 24.23 ± 3.78 kg/m^2^, body weight: 77.21 ± 17.37 kg, body height: 177.71 ± 7.53 cm) were used^27^.

### Measurements

The length and circumference of the upper arm were measured. At rest, the lengths of BB muscle at 60°, 90°, 120°, 150°, and 180° elbow joint positions were measured by marking the proximal and distal ends with the help of ultrasound. The BB muscle’s cross-sectional area (CSA) was calculated using B-mode ultrasound (US) images.

**Figure 6:**
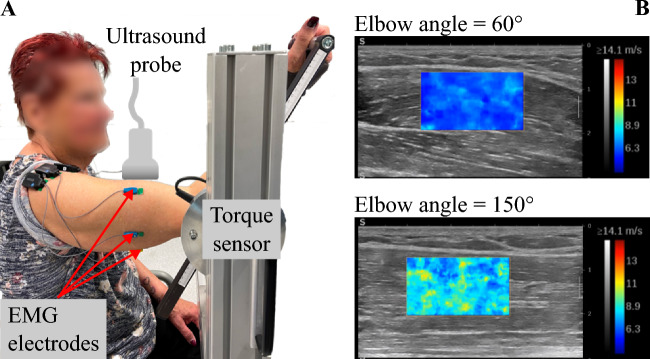
(**A**) Experimental setup. The participant’s elbow joint axis is aligned with the rotational axis of the torque sensor. Surface electromyography (sEMG) electrodes are positioned at the biceps brachii and triceps brachii muscles. The ultrasound probe is placed on top of the biceps brachii muscle belly and aligned with the muscle fiber direction. (**B**) Exemplary ultrasound frames including shear wave propagation speed color overlays during 50% isometric ramp contractions for two of the elbow angles (60°, 150°) studied.

The isometric elbow torque data were collected using a custom-made apparatus with an incorporated torque cell that allows fixing elbow angles at designated positions (Fig. 6A) for details see^27^. The electrical activity of BB and triceps brachii muscle (TB) was measured by placing two dry-surface electrodes^66^ on the long and lateral head of the TB and two electrodes on the BB (Delsys Europe, Greater Manchester, UK) that were slightly medial and lateral to the central position leaving space for US probe (Fig. 6A).

Shear wave propagation velocity was measured using an ultrasonic scanner with SWE and a linear transducer (AixPlorer Mach30, MSK preset; 5–18 MHz, 50mm wide; L18-5; Supersonic Imagine, Aix-en-Provence, France). The transducer was aligned with the muscle fiber direction of BB and placed on the mid-belly (Fig. 6A). The shear elastic modulus G was calculated from the shear wave velocity assuming transverse isotropic and linear elastic material properties for muscle tissue in an unloaded condition as:

1 $${\text{G}} =\uprho \cdot {\text{v}}_{{\text{s}}}^{2}$$

where ρ is the mass density (with ρ_muscle_ ≈ 1.0 kg/m^3^). The shear wave velocity maps were recorded at 1.0–1.8 Hz depending on the size and position of the region of interest (ROI). The maximum shear wave speed provided by the system was 14.1 m/s. sEMG and torque signals were recorded via a data acquisition system (cDAQ-9174, National Instruments, Austin, TX, USA) and sampled with 2 kHz. The synchronous data acquisition and visualization were controlled with a custom MATLAB (The MathWorks, Inc., Natick, USA) script.

### Experimental protocol

Simultaneous measurements of elbow torque, surface electromyography (sEMG), and ultrasound videos (B-Mode and shear wave velocity maps, Fig. 6B) were performed on the subject’s dominant arm. Initially, a warm-up protocol consisting of one elbow flexion maximum voluntary contraction (MVC) and isometric submaximal ramp contractions (from rest, slowly increasing the force production up to 25%, 50%, and 75% MVC torque) at 120° and 180° elbow angles was applied. Starting from the flexed joint position at 60°, passive (at rest) and active (isometric MVC and submaximal ramp contractions) measurements were performed at five elbow angles with 30° intervals up to the fully extended position at 180°. At rest, subjects were asked to relax their muscle as much as possible. To perform the ramp contractions, participants were given visual feedback to follow a trapezoid that consisted of (I) 3 s rest, (II) a ramp up to 25%, 50%, or 75% MVC torque (with 6.25% per second slope), (III) 5 s constant level, (IV) a ramp down to 0% MVC torque (with 6.25% per second slope) and (V) 3 s rest. Two recordings were performed for each condition at each position. Elbow extension MVC torque was measured at 120° elbow angle twice. To prevent fatigue or potential stretching effects,^67^ muscles were rested for at least one minute following MVCs and 30 s after ramp contractions.

### Data analysis

The maximum elbow torque was calculated for each position from the MVC trials. For the submaximal ramp contractions, the mean torque was calculated for the middle three seconds of part III. The mean absolute percentage deviation (MAPD) of applied torque was calculated over the part III of the trapezoid. Trials above 10% MAPD were excluded from further analysis (including sEMG and SWE) to minimize the performance or concentration errors of the participants.

The sEMG data were filtered (fourth-order butter-worth filter, 20–350 Hz bandwidth) and full-wave rectification was applied. As a measure of the sEMG amplitude, the root mean square moving average of 250 ms windows was calculated. The averages of the signals collected from the two BB electrodes and two TB electrodes were used as representatives of the sEMG of BB and TB, respectively. For all trials, the mean amplitude was calculated from the middle three seconds of the measurement. The MVC sEMG amplitude at each elbow angle was used for the normalization of the resting trials. For the normalization of the TB sEMG, the MVC extension trial at 120° elbow angle was used. sEMG amplitudes of ramp contraction trials were normalized to the average amplitude during the constant level (part III) of the 75% ramps for both BB and TB.

Color analyses of the SWE videos were performed by defining the ROI manually covering only muscle fibers and calculating the mean shear elastic modulus. The recordings were analyzed frame by frame. If more than 25% of the pixels inside the ROI were missing, no result was assigned to that frame. For analysis of the rest and MVC, the mean shear elastic modulus was calculated from all available frames. Ramp contraction recordings were resampled to 1 Hz from their original SWE sampling rate and mean shear elastic modulus was calculated from the middle three seconds of the constant level (part III). The active shear elastic modulus was calculated for MVC and ramp contraction trials by subtracting the respective passive shear elastic modulus from the measured (total) shear elastic modulus.

Participants’ resting state was evaluated by calculating the number of trials at rest where the sEMG amplitude of the BB was above 1%, 3%, and 5% after normalized to their maximum values at MVC. 3% was used as an exclusion criterion for the evaluation with the assumption that they may not represent the muscle’s passive state. After excluding individual trials above thresholds, the two trials collected at the same condition were averaged.

To compare the performance of the older group in following the intended torque level with the young group^27^, the MAPD values calculated for parts II-IV of the trapezoid were used. To determine the effect of elbow angle, muscle contraction intensity level, and age on the measurands, analysis of variation (ANOVA) with a significance level of α = 0.05 was performed. Pairwise comparison tests were performed with the Bonferroni method as post hoc analyses.

### Institutional review board statement

The study was conducted in accordance with the Declaration of Helsinki, and approved by the Institutional Review Board (or Ethics Committee) of the University of Tübingen (protocol code 612/2021BO2 from 15.09.2021 and 22.10.2021).

### Informed consent

Informed consent was obtained from all subjects involved in the study.

## Data Availability

The data presented in this study are available on request from the corresponding author.
